# Rostral Anterior Cingulate Thickness Predicts the Emotional Psilocybin Experience

**DOI:** 10.3390/biomedicines8020034

**Published:** 2020-02-18

**Authors:** Candace R. Lewis, Katrin H. Preller, B. Blair Braden, Cory Riecken, Franz X. Vollenweider

**Affiliations:** 1 Translational Genomics Research Institute, Neurogenomics Division, Phoenix, AZ 85004, USA; 2 Neuropsychopharamacology and Brain Imaging, Department of Psychiatry, Psychotherapy and Psychosomatics, University Hospital for Psychiatry, Zurich 8032, Switzerland; preller@bli.uzh.ch (K.H.P.); vollen@bli.uzh.ch (F.X.V.); 3 Arizona State University, College of Health Solutions, Tempe 85281, AZ 85004, USA; bbbraden@asu.edu (B.B.B.); criecken@asu.edu (C.R.)

**Keywords:** psilocybin, 5HT2Ar, cingulate, emotion

## Abstract

Psilocybin is the psychoactive compound of mushrooms in the *psilocybe* species. Psilocybin directly affects a number of serotonin receptors, with highest affinity for the serotonin 2A receptor (5HT-2Ar). Generally, the effects of psilocybin, and its active metabolite psilocin, are well established and include a range of cognitive, emotional, and perceptual perturbations. Despite the generality of these effects, there is a high degree of inter-individual variability in subjective psilocybin experiences that are not well understood. Others have shown brain morphology metrics derived from magnetic resonance imaging (MRI) can predict individual drug response. Due to high expression of serotonin 2A receptors (5HT-2Ar) in the cingulate cortex, and its prior associations with psilocybin, we investigate if cortical thickness of this structure predicts the psilocybin experience in healthy adults. We hypothesized that greater cingulate thickness would predict higher subjective ratings in sub-scales of the Five-Dimensional Altered State of Consciousness (5D-ASC) with high emotionality in healthy participants (*n* = 55) who received oral psilocybin (either low dose: 0.160 mg/kg or high dose: 0.215 mg/kg). After controlling for sex, age, and using false discovery rate (FDR) correction, we found the rostral anterior cingulate predicted all four emotional sub-scales, whereas the caudal and posterior cingulate did not. How classic psychedelic compounds induce such large inter-individual variability in subjective states has been a long-standing question in serotonergic research. These results extend the traditional set and setting hypothesis of the psychedelic experience to include brain structure metrics.

## 1. Introduction

In 1953, relatively high concentrations of serotonin (5-hydroxytryptamine or 5-HT) were found in the brain [1]. Five years later, the Swiss chemist Albert Hofmann isolated psilocybin (4-phosphor yloxy-N,N-dimethyltryptamine), an indole alkaloid of the tryptamine family, from mushrooms of the genus Psilocybe used for ceremonial purposes in Mexico. Psilocybin is rapidly dephosphorylated into the psychoactive metabolite psilocin (4-hydroxy-*N,N*-dimethyltryptamine) in vitro [2] and in vivo [3]. Pharmacological studies in rats have shown that psilocin primarily binds to 5-HT2A receptors (5-HT2Ar), with a lower affinity for the 5-HT1Ar and several other 5-HT receptor subtypes [4,5,6]. Generally, the effects of psilocybin, and its active metabolite psilocin, on human behavior are well established and include a range of cognitive, emotional, and perceptual perturbations [7,8,9,10,11,12,13,14,15,16,17]. Studies using a preferential 5-HT2Ar antagonist have demonstrated psilocybin’s psychoactive effects are primarily mediated through this receptor [18].

Despite the generality of the psilocybin experience, there is also a high amount of inter and intra-individual variability in psilocybin subjective responses. Psychedelic research of the 1960s attributed variability in drug response with the concept of set and setting (for review see [19]). The set and setting principle posits that the psychedelic experience is highly determined by the extra-pharmacological parameters of set (personality, preparation, expectation, and intention) and setting (the physical, social, and cultural environment). Our group and others have postulated and demonstrated the relative importance of set and setting variables for the subjective response to psychedelics for over half a century [20,21,22,23]. For example, we previously used a pooled analyses of 261 individuals to test 24 non-pharmacological variables as predictors of the subjective effects of psilocybin and found, indeed, a variety of set and setting variables are associated with many aspects of the psilocybin response in healthy volunteers [22]. However, there is a lack of research specifically assessing biological drivers of the psilocybin experience.

Individual brain morphology measures can be used to predict various pharmacological challenges and behavior. For example, thicker frontal cortices predict less D-amphetamine induced striatal dopamine release measured by positron emission tomography (PET) [24,25,26]. Brain structure metrics are associated with several aspects of depression such as risk factors, neurobiological correlates, and pharmacological treatment response [27,28,29,30]. Furthermore, individual differences in the personality dimensions of novelty seeking, harm avoidance, reward dependence, and persistence also reflect structural variance in specific brain regions [31,32,33]. Therefore, it is reasonable to presume individual differences in brain morphologic measures may predict the individual subjective experience of psilocybin.

**Figure 1:**
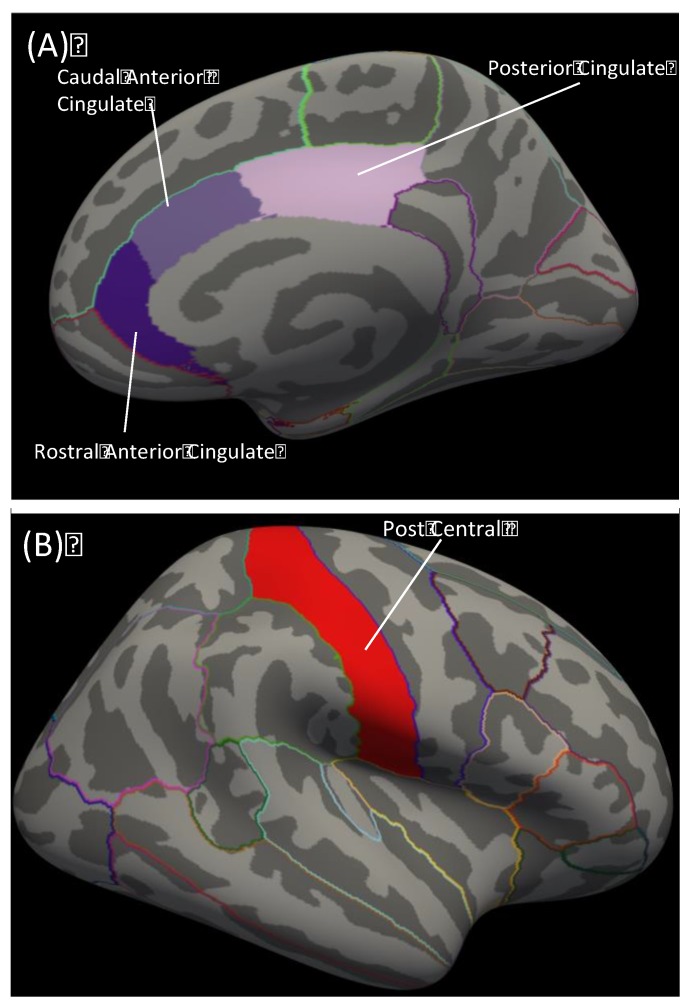
Schematic of FreeSurfer brain regions used in analyses. (**A**) Right hemisphere cingulate cortex parcellations used in primary analyses. Dark purple = rostral anterior cingulate; medium purple = caudal anterior cingulate; light purple = posterior cingulate. (**B**) Red = right hemisphere post central parcellation used as the control region analysis.

In addition to cognitive and perceptual distortions, the effects of psilocybin on emotionality have been a growing point of interest due to the psycho-therapeutic potential of these compounds. Therefore, we aimed to identify morphological features that predict the subjective emotional experience of psilocybin. The Five-Dimensional Altered States of Consciousness Scale (5D-ASC) is a reliable and validated tool often used to measure the subjective psilocybin experience [34,35]. The 5D-ASC comprises 11 subscales derived from questions assessing the subjective experiences of an altered state of consciousness in retrospect [34]. Of the 11 5D-ASC constructs, the four constructs with high emotionality are: Feeling of Unity, Bliss, Spiritual Experience, and Insightfulness. Psilocybin modulates activity and functional connectivity throughout the limbic system, which is considered the central emotional network [13,16,17,36,37]. Because the subjective effects of psilocybin are primarily mediated by the 5-HT2Ar [18], we chose regions of the limbic system with the highest 5-HT2Ar protein expression, and identified the cingulate cortex based on prior research (Figure 1A) [38]. We hypothesized that greater cingulate thickness would predict higher subjective ratings in healthy participants (*n* = 55) who received psilocybin (either low dose: 0.160 mg/kg or high dose: 0.215 mg/kg).

## 2. Materials and Methods

### 2.1. Participants

Participants were recruited through advertisements in local universities for two separate groups, either low dose (0.16 mg/kg: Low) or a high dose (0.215 mg/kg: High). All participants were healthy based on medical history, physical examination, blood analysis, and electrocardiography, had normal or corrected-to-normal vision, and were urine tested for drug abuse and pregnancy. The combined data used for this analysis included 55 participants (33 males, 22 females; mean age 25, SD 3.96 years, range 20–37 years). Participants received written and oral descriptions of the study procedures as well as the effects and possible risks of psilocybin administration. All participants provided written informed-consent statements in accordance with the declaration of Helsinki before participation in the study. The Swiss Federal Office of Public Health, Bern, Switzerland authorized the use of psilocybin in humans. Furthermore, the Cantonal Ethics Committee of Zurich approved the study (KEK 2014-0496 12-12-2014; KEK 2012-0303 13-09-12).

### 2.2. Experimental Design and Psilocybin Administration

In a randomized, double blind, and placebo-controlled study, subjects received either placebo (maltose) or oral psilocybin (0.16 mg/kg or 0.215mg/kg) in two separate sessions at least 10 days apart. These doses reflect the low and high end of the medium dose range commonly used in the literature [12,39]. Participants had to abstain from smoking for at least 60 min before MRI assessments and from drinking caffeine during the test day. An anatomical scan was conducted 60 min after drug administration. The 5D-ASC [34], a self-report questionnaire retrospectively assessing the subjective experience after drug intake, was completed 360 min after intake. The 5D-ASC comprises 94 items to be answered on visual analogue scale. Scores have been validated for 11 sub-scales [35], of which we used four in this study: experience of unity (example item: “I experienced past, present, and future as a oneness”), spiritual experience (example item: “I experienced a kind of awe”), blissful state (example item: “I enjoyed boundless pleasure”), and insightfulness (example item: “I gained clarity into connections that puzzled me before”). All items that comprise these subscales can be found here [35]. Participant report of psilocybin minus placebo was used to calculate the delta (Δ) variables used in analyses.

### 2.3. Neuro-Imaging Acquisition

All MR data were acquired on a Philips Achieva 3.0T whole-body scanner (Best, The Netherlands). Inflatable pillows (Multipad, Pearltec AG, Zurich, Switzerland) were used to increase participant comfort in the scanner and to reduce motion induced artifacts. High-resolution anatomical images (voxel size, 1 × 1 × 1 mm) were acquired using a standard T1-weighted three-dimensional (3D) magnetization prepared rapid gradient echo sequence (MP-RAGE). Each session consisted of a resting state arterial spin labeling perfusion-weighted scan and several task-related blood-oxygen-level-dependent scans [13,16,17].

### 2.4. Image Data Processing

T1-weighted images from the placebo session were processed to obtain cortical thickness using FreeSurfer version 6.0 image analysis suite (surfer.nmr.mgh.harvard.edu/). These processing steps have been described in detail elsewhere [40,41,42]. Cortical thickness was defined as the distance between the white/gray matter boundary to the pial surface. We chose cortical thickness, rather than surface area or volume, because of previous literature associating this metric with drug response [24,25,26]. Segmentations were visually inspected to ensure accuracy of automated processes. Regions of interest were based on high 5HT-2Ar expression in limbic regions (rostral anterior cingulate, caudal anterior cingulate, and posterior cingulate; Figure 1) [38]. A control region was chosen on lowest 5HT-2Ar expression in a cortical region (post central; Figure 1B) [38]. Cortical thickness values for the three areas of the cingulate and the post central gyrus were extracted from the Desikan atlas.

### 2.5. Statistical Analyses

To assess a difference in the sub-scales between dose groups we ran an analysis of covariance (ANCOVA), controlling for sex and age. Because there was no difference between groups, for further analyses we collapsed across dose and used it as a covariate. We ran multivariate linear regressions controlling for sex, age, control region, and dose with rostral anterior cingulate, caudal anterior cingulate, and posterior cingulate thickness as predictor variables for all models for the four emotional sub-scales. The 5D-ASC sub-scale variables were calculated as psilocybinScale – placeboScale = ΔScale. The Benjamini-Hochberg (false-discovery rate; FDR) procedure was conducted to control for multiple comparisons and Type 1 error within each hemisphere. Lastly, as a follow up to the significant associations, we compared the correlated correlation coefficients between the three cingulate regions in the right hemisphere for each sub-scale to determine if they were significantly different using methods outlined here [43].

## 3. Results

### 3.1. Dose Comparison

There were no significant differences between the two doses on the behavioral sub-scales (Table 1). Therefore, for further analyses we collapsed across dose and included dose as a covariate.

### 3.2. Cingulate Thickness Predicting Sub-Scales

**Figure 2:**
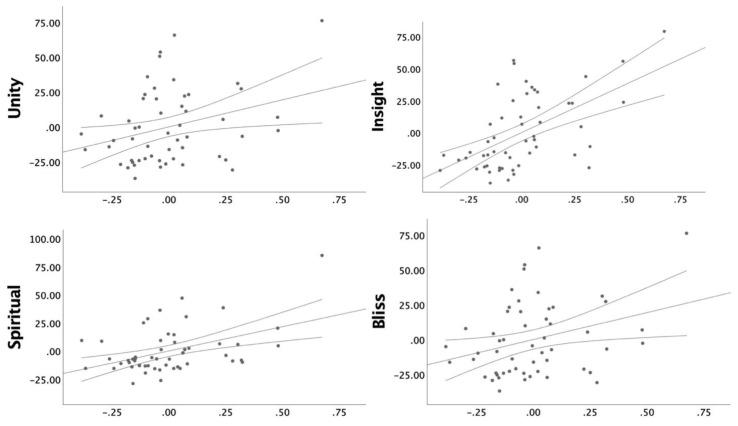
Partial correlation plots controlling for sex, age, and dose between responses to psilocybin (Unity, Bliss, Spiritual, and Insight) and estimates of right hemisphere rostral anterior cingulate cortex (R rACC) thickness with 95% confidence intervals. Scatter plots for non-significant results can be found in Supplementary Materials. Axes represent normalized values. Response to psilocybin is represented as the delta value; psilocybin – placebo.

We found a significant positive association between the right hemisphere rostral anterior cingulate thickness and all ΔScales; Feeling of Unity, Bliss, Spiritual Experience, and Insightfulness (Figure 2). Standardized beta values, SE, p, and FDR corrected *p* values can be found for all the rostral anterior cingulate models in Table 2. We found no significant associations with the caudal anterior cingulate or posterior cingulate cortex thickness and ΔScales (Table 3).

### 3.3. Comparing Correlated Correlation Coefficients

We found the correlation coefficients between right hemisphere rostral anterior, caudal, and posterior cingulate for Unity (*p* = 0.05), Spiritual (*p* < 0.001), Insight (*p* < 0.001), and Insight (*p* < 0.001) were significantly different using methods outlined here [43].

## 4. Discussion

This is the first study to evaluate brain morphology as a predictor of the emotional subjective experience of psilocybin in healthy controls. We focused our analyses on the cingulate cortex based on high 5-HT2Ar expression levels compared to other limbic regions. We found rostral anterior cingulate thickness specifically predicted all four sub-scales of Unity, Spiritual Experience, Blissful State, and Insightfulness, whereas the caudal anterior and posterior cingulate did not. These results point towards the possibility that morphology metrics such as cortical thickness may reflect differences in brain substrates that mediate drug effects.

The anterior portion of the cingulate is a unique region of the brain for its connections to both the limbic system and the more cognitive prefrontal cortex [44]. Our results of rostral, but not other sub-divisions of the cingulate, predicting emotional experiences aligns well with current anatomical knowledge of the cingulate. Briefly, the anterior cingulate has extensive connections with areas known to be important for emotion, memory, and reward related functions whereas the caudal cingulate has extensive connections with cognitive and motor-related areas of cortex [41]. Lastly, the posterior cingulate has connections throughout the neocortex, is crucial component of the default mode network, and is thought to play a pivotal role in the recall of autobiographical memories [45]. While it was surprising to us that posterior cingulate thickness did not predict the psilocybin emotional experience due to the structure’s previous associations with psilocybin [46], our results further highlight the importance of anterior cingulate in emotional processing. For example, others have found the anterior cingulate to be involved in emotional and self-processing in healthy controls [47,48], and anterior cingulate activity changes have been associated with Mindfulness-Based Stress Reduction interventions in back pain patients with depression [49]. While the amygdala is typically considered the key limbic structure with a central role in emotion [50], emotional control is considered a “top-down” regulation process from several areas of frontal cortex [51]. For example, the anterior cingulate cortex projects to both the amygdala and the prefrontal cortex, which is thought to provide the capacity for emotional regulation [52]. Taken together, one might speculate that cingulate 5-HT2A activation by psilocybin/psilocin plays a major role in inducing the profound emotional occurrences often associated with psilocybin.

There is some evidence suggesting the experience of emotion (i.e., mood and affect) is predominantly regulated by the right hemisphere [53,54]. Our results indicate right hemisphere cingulate morphology is a better predictor of psilocybin-induced emotional experiences compared to the left hemisphere. These results align well with the previous findings from our group and others that classic psychedelic compounds induce hyperfrontal effects particularly in the right hemisphere [9,13,55,56,57]. Right-hemisphere processing is historically described to possess holistic, gestalt, or integrative characteristics compared to the left hemisphere [58,59]. Specifically, the right hemisphere is superior in processing emotional affect and affective linguistic characteristics such as metaphor [53,60]. Because the right hemisphere appears to be involved in emotional processing, perhaps the right lateralized effects of psilocybin play a role in its reported psychotherapeutic effectiveness across a wide range of psychiatric disorders [61].

A recent open-label trial using psilocybin for treatment resistant depression found the composite variable of Unity, Spiritual Experience, and Blissful State along with Insightfulness predicted therapeutic outcomes [62]. The spiritual experience of psilocybin, along with the persistent increased sense of well-being and life satisfaction, has been reported to account for sustained reductions in smoking and drinking behavior and decreased depression and anxiety [39,63,64,65,66,67]. While the underlying mechanism(s) linking these subjective states with long-term reductions in maladaptive behavior are unknown, these emotional states reflect common therapeutic goals such as decreasing social disconnection, negative rumination, and hopelessness [68,69,70,71,72]. Furthermore, it is hypothesized that the direct 5-HT2Ar agonist properties of psilocybin enhance sensitivity and facilitates emotional release, which, when combined with psychological support, leads to symptom reduction [73]. If psilocybin clinical trials continue to demonstrate effective therapeutic properties, discovering individual biomarkers to help predict which patients are most likely to benefit from these therapies will be an important line of research [74]. Biomarkers prospectively predicting effective treatment options could assist in individualized treatment planning, reducing time spent on ineffective treatments, shortening patient suffering, and reducing the overall cost burden of depression [75]. Our results suggest neuroimaging may benefit precision medicine approaches of future psychedelic assisted treatment options.

One limitation of this study is that our design does not allow for a causal inference between brain morphology and psilocybin subjective states. However, our self-report data does take into account the participant’s baseline emotional states by using self-report from both psilocybin and placebo sessions. This is important as it allows us to correlate cingulate thickness with psilocybin-induced states while functionally controlling for variability in participant baseline mood, excitement, or study stress. While including two separate doses of psilocybin is a strength, it is also a limitation that doses were both in the medium range; thus, we did not detect dose effects. Another strength of this study is the use of modern neuroimaging methods to extend the set and setting hypothesis to include in vivo individual brain structure information. However, future research should also include genomic and epigenetic predictors of individual psilocybin response since allelic variations in monoaminergic genes have been linked to brain structure and therapeutic response to pharmacological treatments [76,77,78,79,80,81]. Lastly, because we assessed these relationships with a healthy sample, future research with patient populations will need to determine if our results prove to be of clinical value.

How classic psychedelic compounds induce such large inter- and intra-variability in subjective states has been a long-standing question in serotonergic research. Set and setting has been the prevailing theory for over half a century. Several studies validated this theory and found significant relationships between non-pharmacological predictors and the psilocybin experience; however, there are still relatively large proportions of unexplained variance in individual emotional experiences [19,22]. Investigating the relationship between psilocybin modulation and cingulate structure could provide insight into mechanisms underlying psilocybin’s profound emotional effects and provides a theoretical framework for brain-based predictors of 5HT2A psychedelic modulation. These results provide the basis for a potential new unified theory of set, setting, and structure as predictors of the individual psychedelic experience.

## Figures and Tables

**Table 1:** Comparison between doses on sub-scales.

	Low (0.16 mg/kg)		High (0.215mg/kg)		*p*-Value
Construct	Mean	SE	Mean	SE	
Unity	26	5	33	5	0.495
Spiritual	12	4	16	4	0.975
Bliss	38	6	43	6	0.75
Insight	25	5	36	6	0.516

Max value = 100. Values reported as psilocybin - placebo.

**Table 2:** Right Hemisphere Rostral Anterior Cingulate Thickness Predicts the Emotional Psilocybin Experience.

Sub-Scale	Rostral Anterior Cingulate Cortex (rACC)			
	*β*	SE	*p*	FDR *p*
*Unity*				
LH	0.295	17.93	0.037	0.114
RH	0.324	17.18	0.027	0.027
*Bliss*				
LH	0.106	25.14	0.465	0.465
RH	0.386	18.95	0.008	0.011
*Spiritual*				
LH	0.266	13.84	0.057	0.114
RH	0.465 *	12.35	0.001	0.002
*Insight*				
LH	0.179	20.26	0.202	0.269
RH	0.572 *#	16.46	0.00002	0.00008

FDR: false discovery rate; LH: left hemisphere; RH right hemisphere; β: standardized regression coefficient; SE: standard error; * Age < 0.05; # Dose < 0.05.

**Table 3:** Caudal and posterior cingulate thickness do not predict psilocybin emotional experience.

Sub-Scale	Caudal Cingulate			Posterior Cingulate		
	*β*	SE	*p*	*β*	SE	*p*
*Unity*						
LH	−0.021	22.383	0.884	0.108	22.289	0.454
RH	0.275	16.976	0.065	0.062	30.219	0.676
*Bliss*						
LH	−0.106	25.141	0.465	−0.046	25.287	0.749
RH	0.006	19.852	0.969	0.01	34.181	0.947
*Spiritual*						
LH	0.213	16.749	0.132	0.052	17.144	0.711
RH	0.109	13.39	0.463	−0.028	23.175	0.852
*Insight*						
LH	0.186	24.158	0.186	0.125	24.428	0.371
RH	0.241	18.781	0.099	−0.033	33.242	0.82

FDR: false discovery rate; LH: left hemisphere; RH right hemisphere; β: standardized regression coefficient; SE: standard error; * Age < 0.05; # Dose < 0.05.

## Supplementary Materials

Supplementary materials can be found at https://www.mdpi.com/2227-9059/8/2/34/s1.
