# Supplementary figures and images for: Rostral Anterior Cingulate Thickness Predicts the Emotional Psilocybin Experience
Source: Biomedicines. 2020 Feb 18;8(2):34. doi: 10.3390/biomedicines8020034 (PMC7168190; doi:10.3390/biomedicines8020034)

Unity

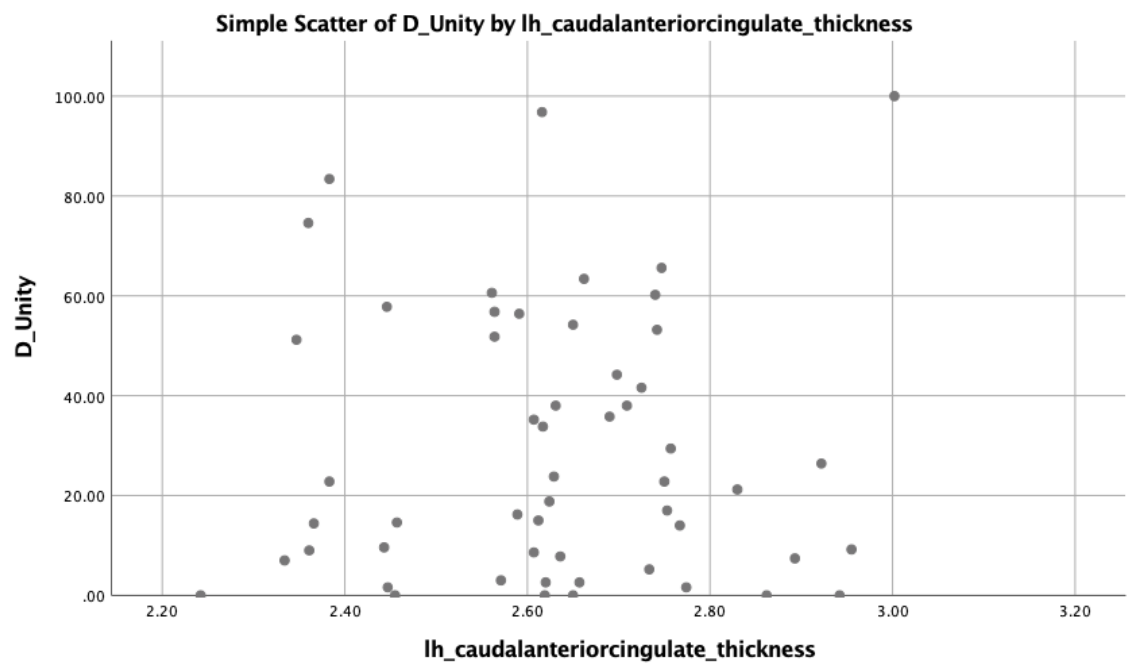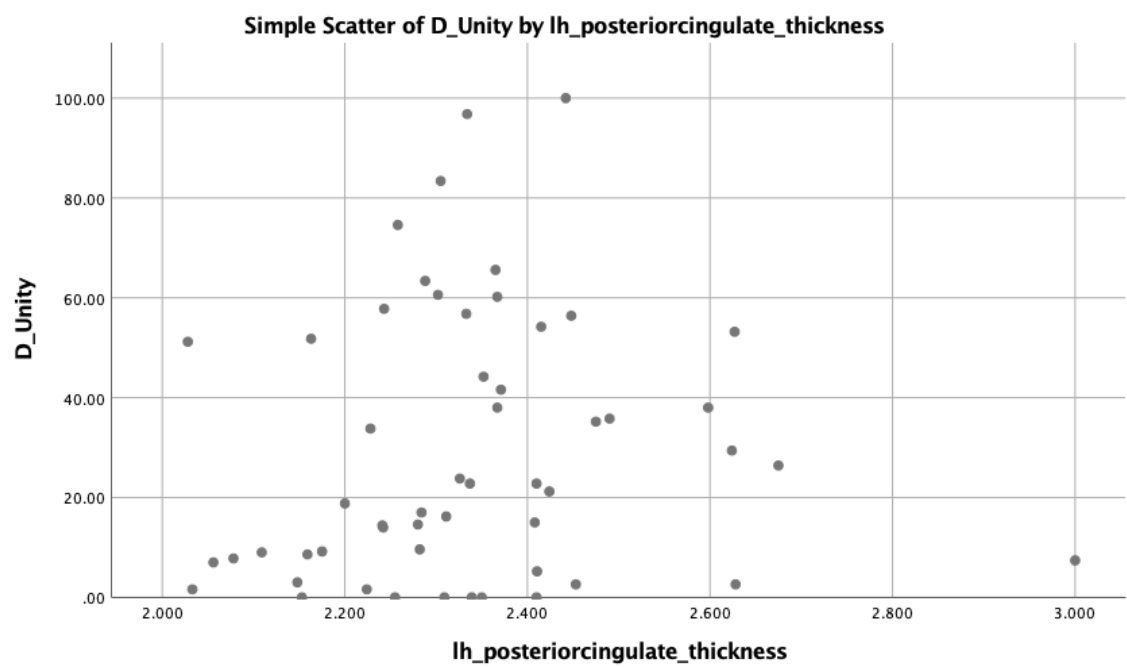

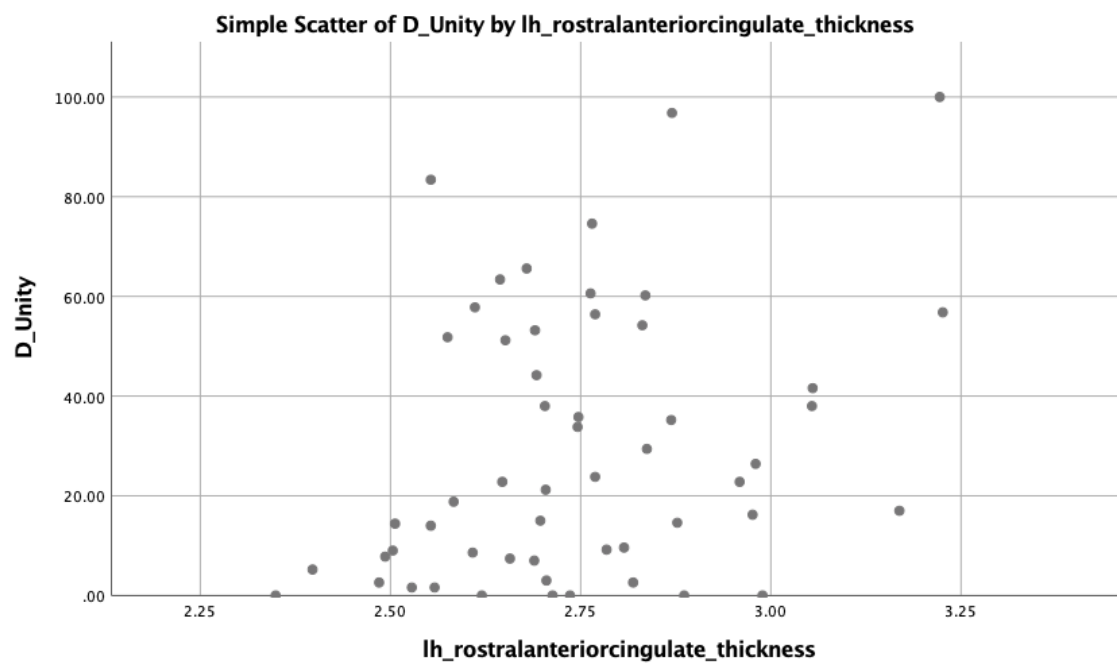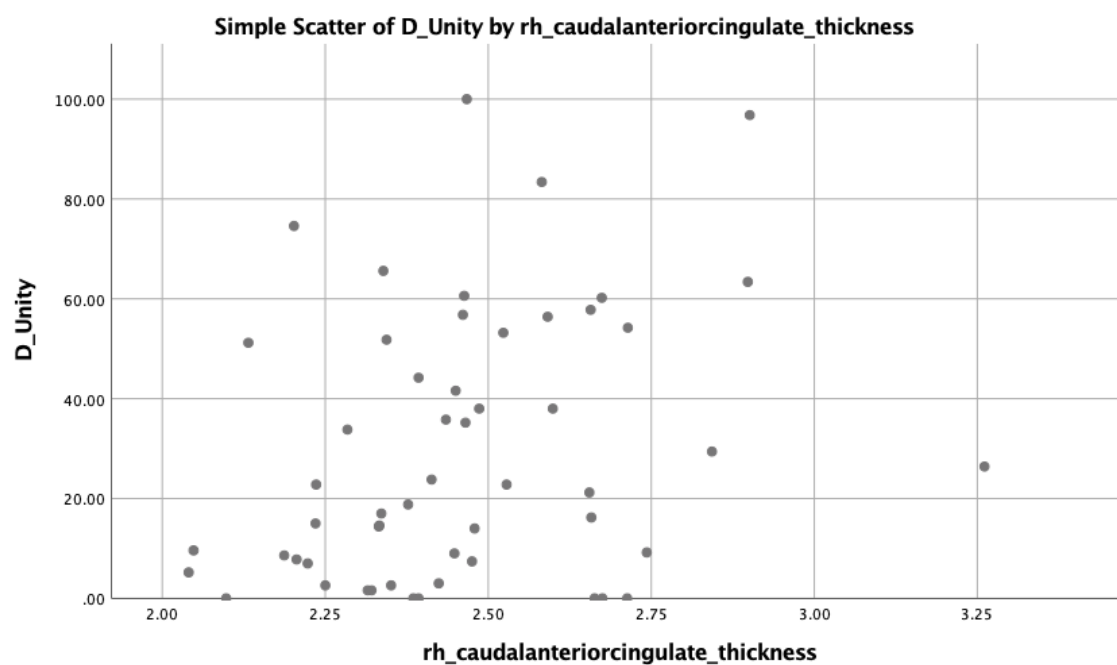

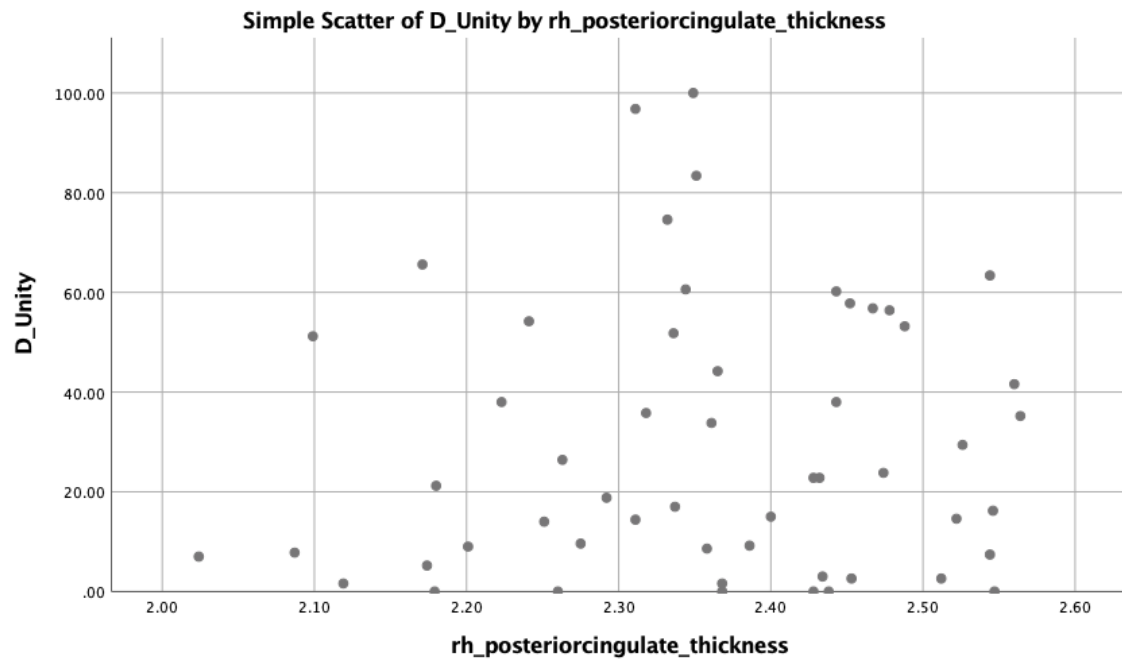

Spiritual Experience

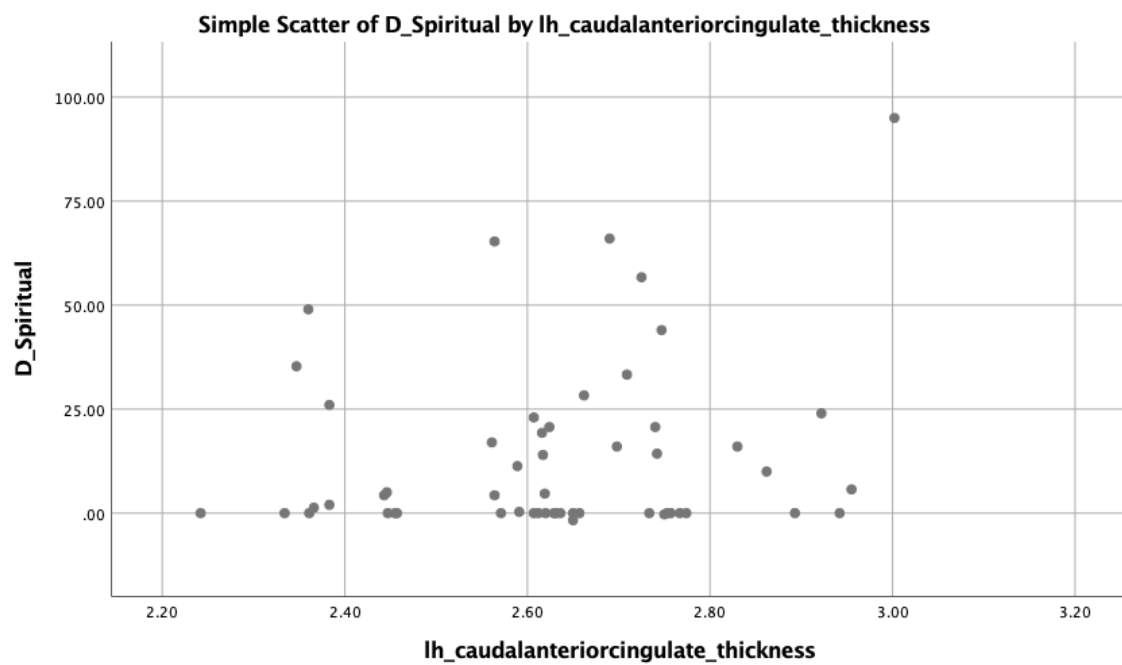

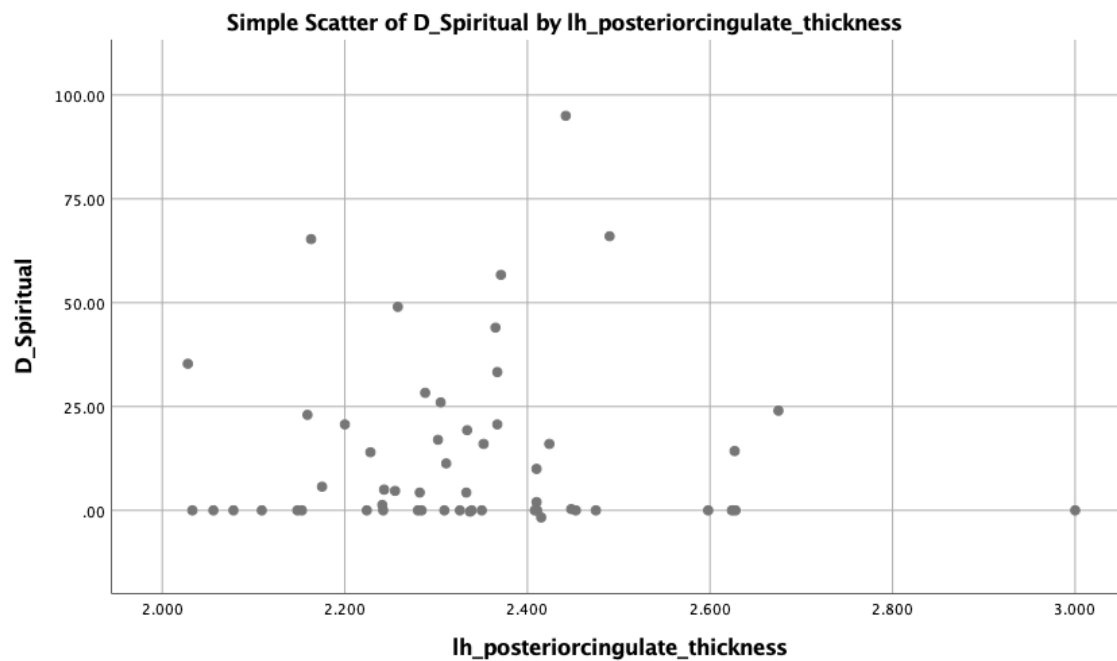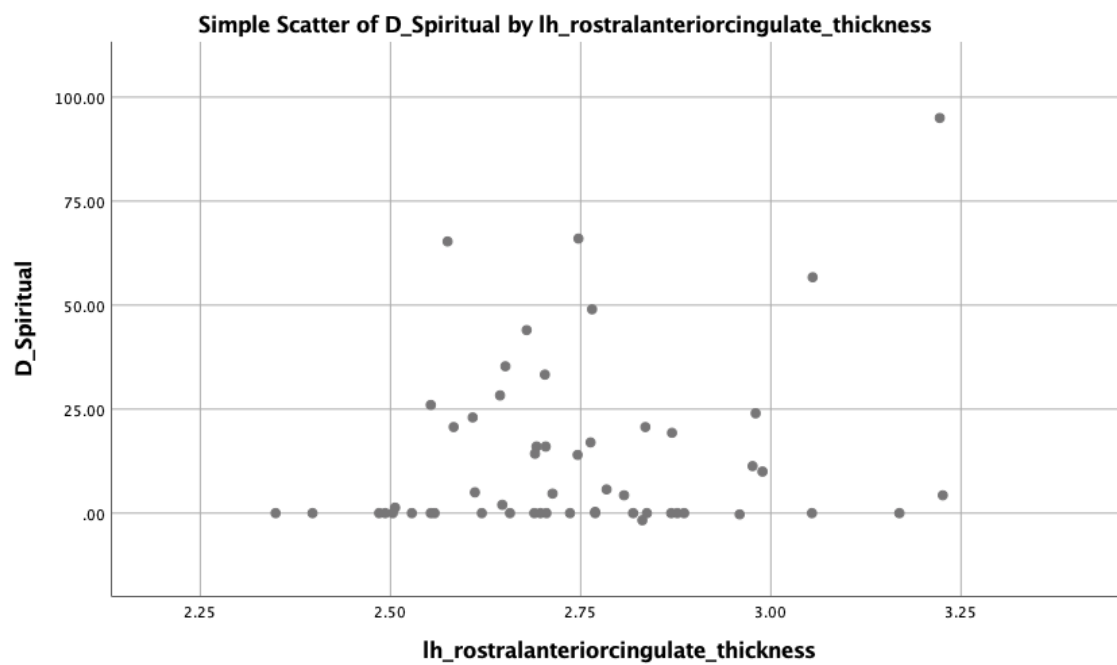

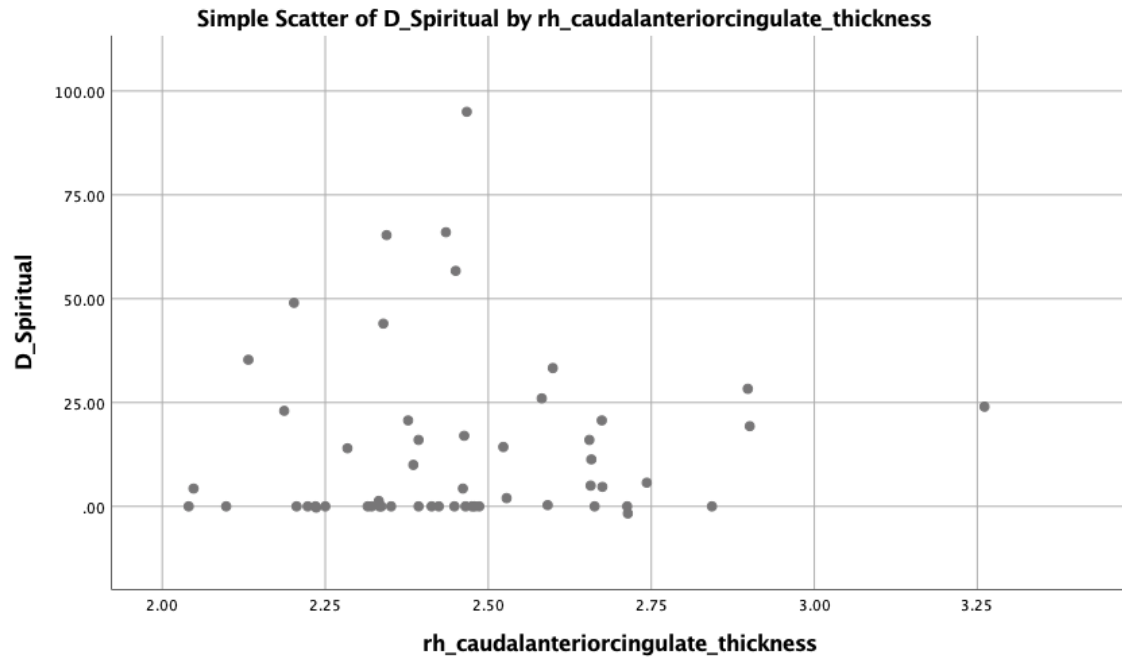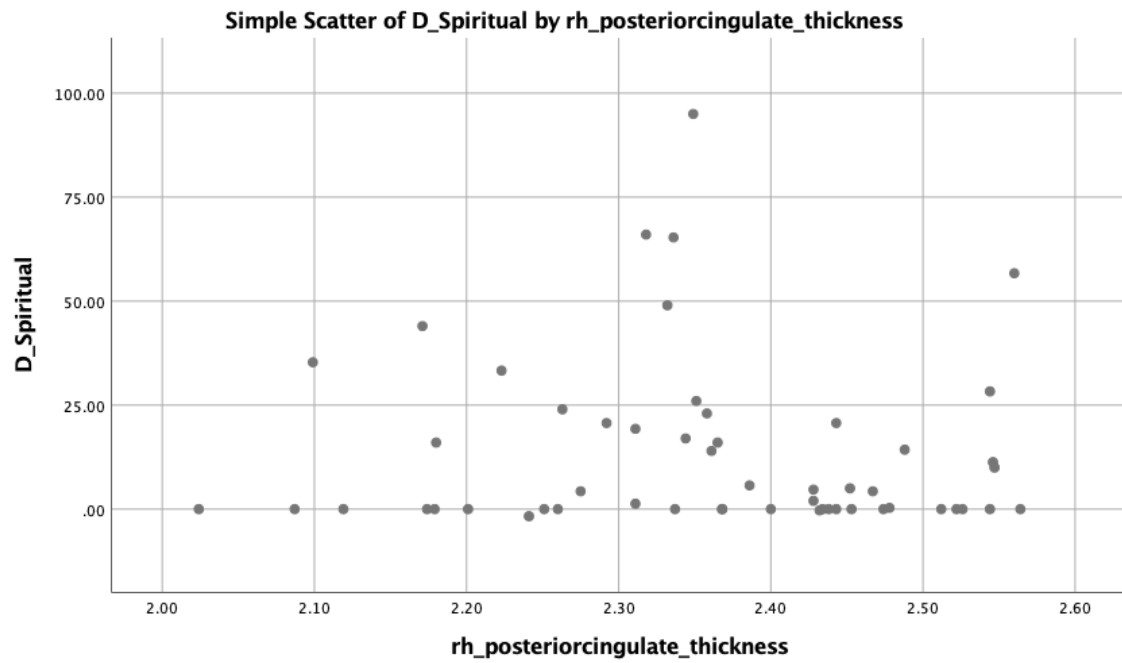

Bliss

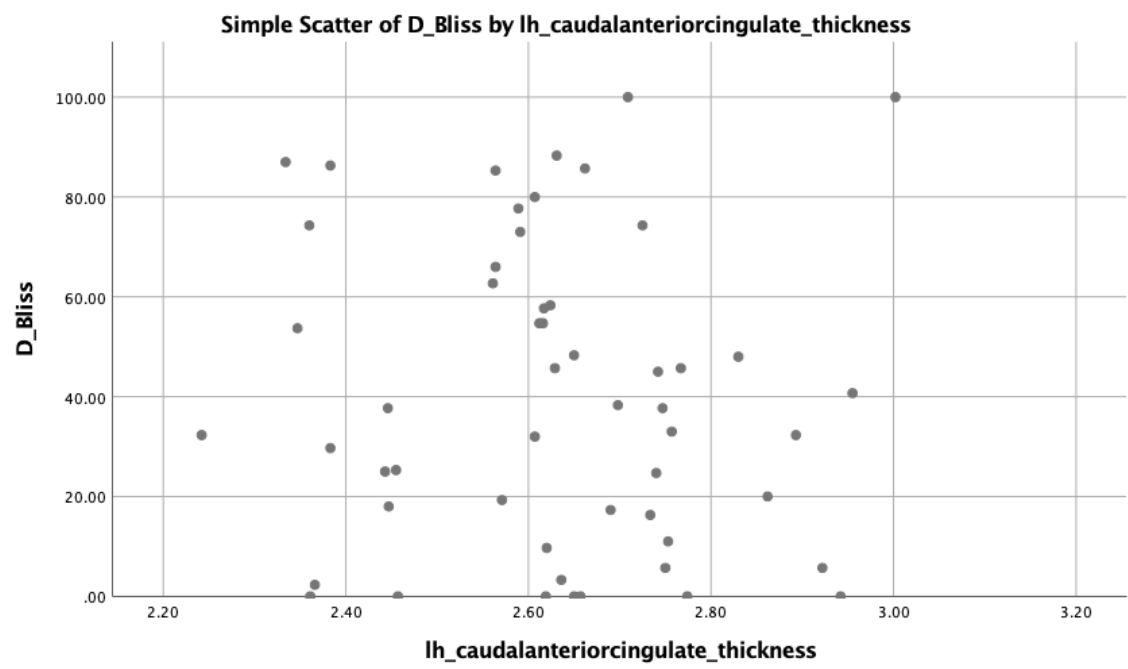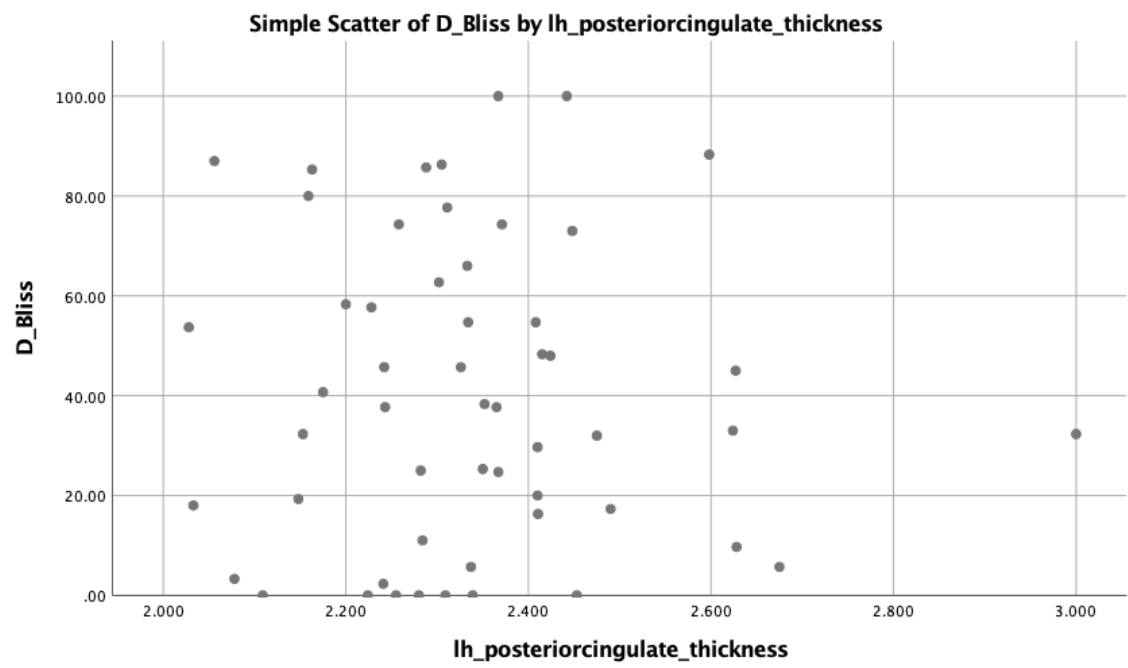

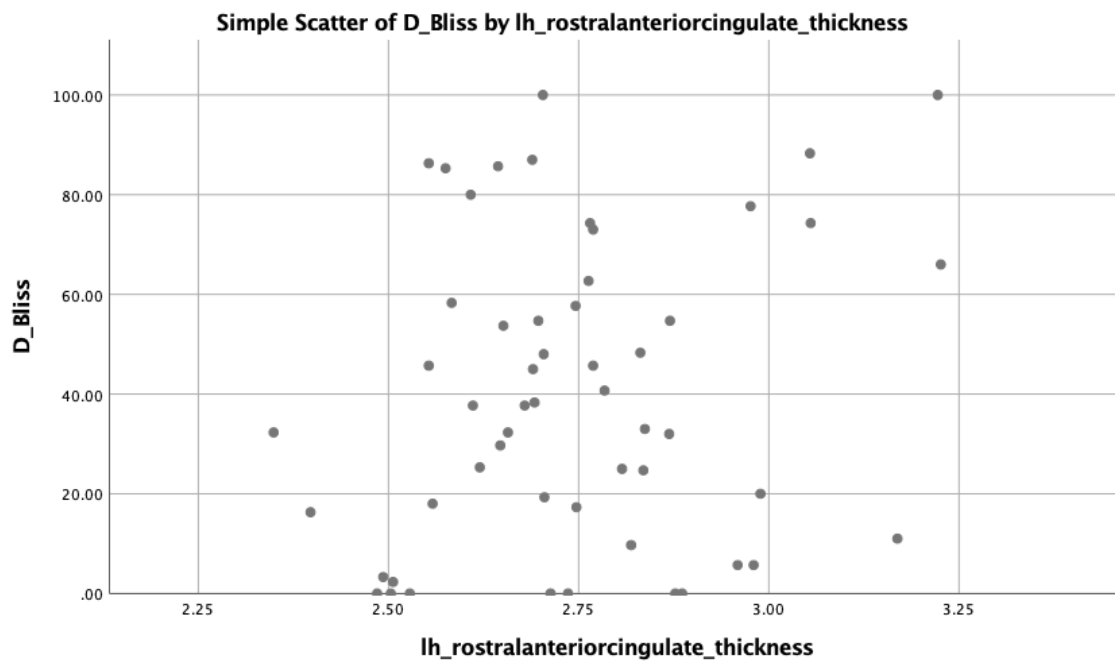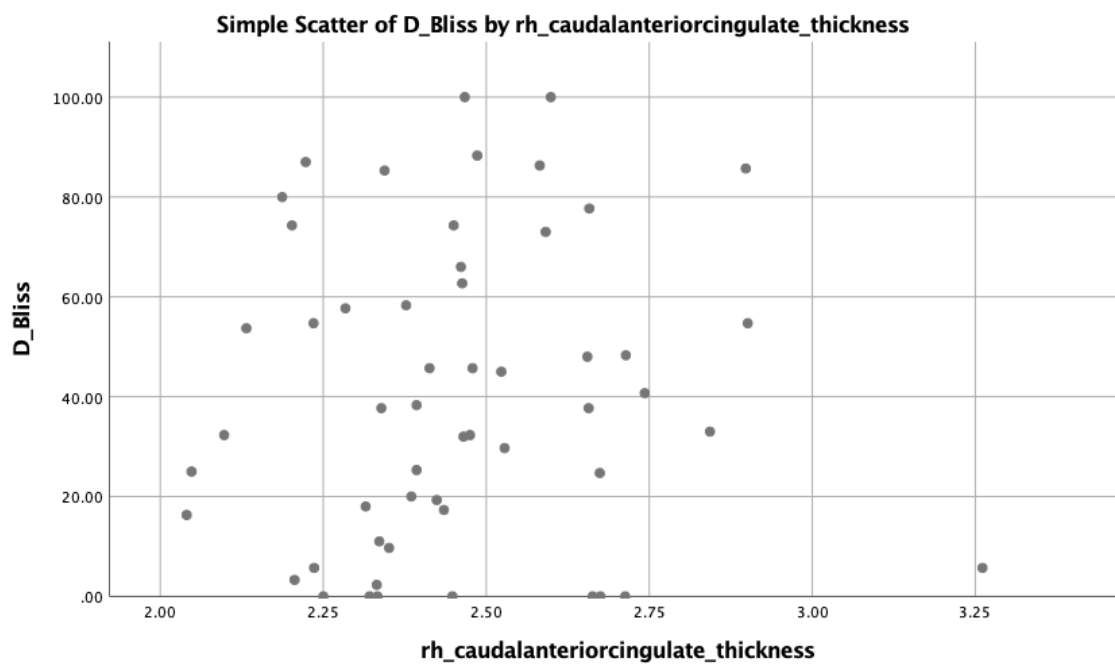

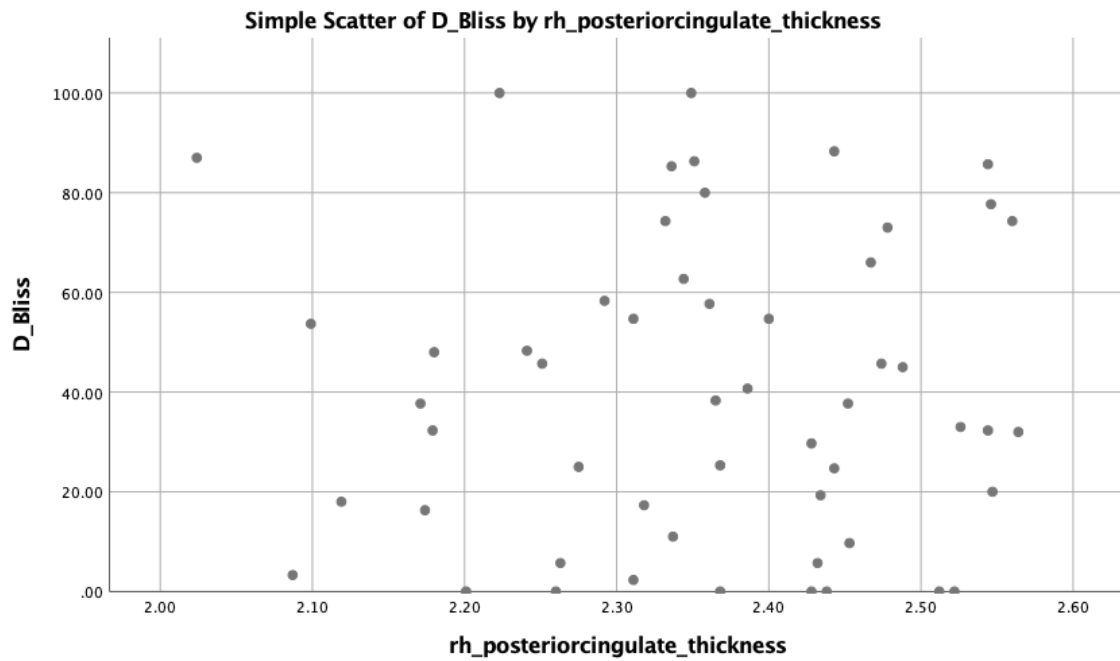

Insightfulness

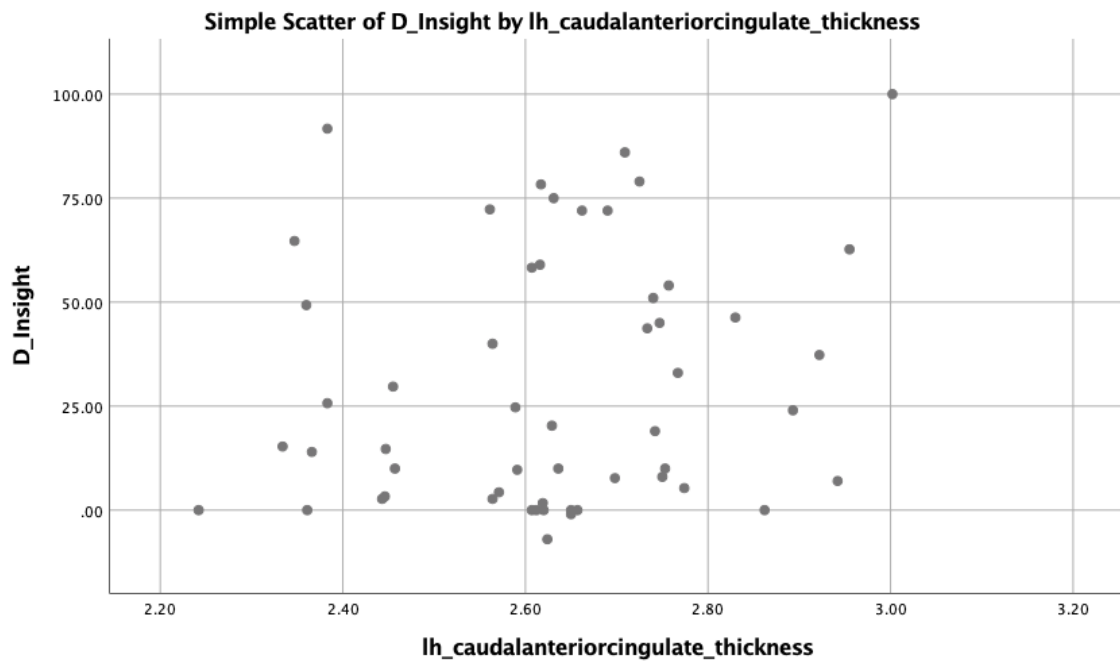

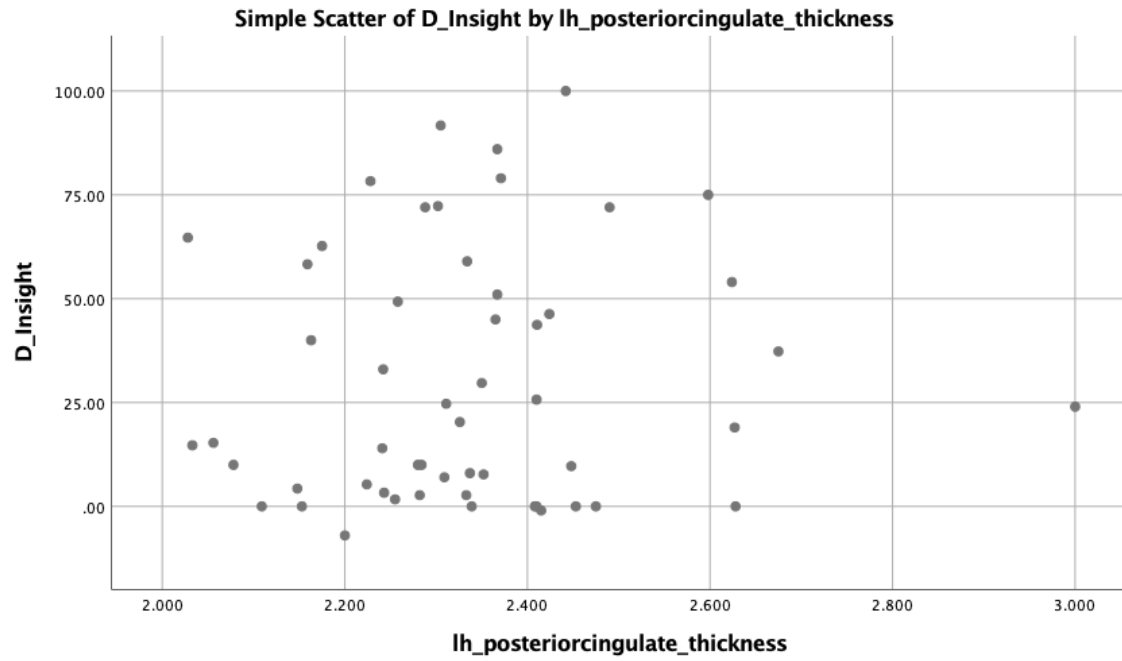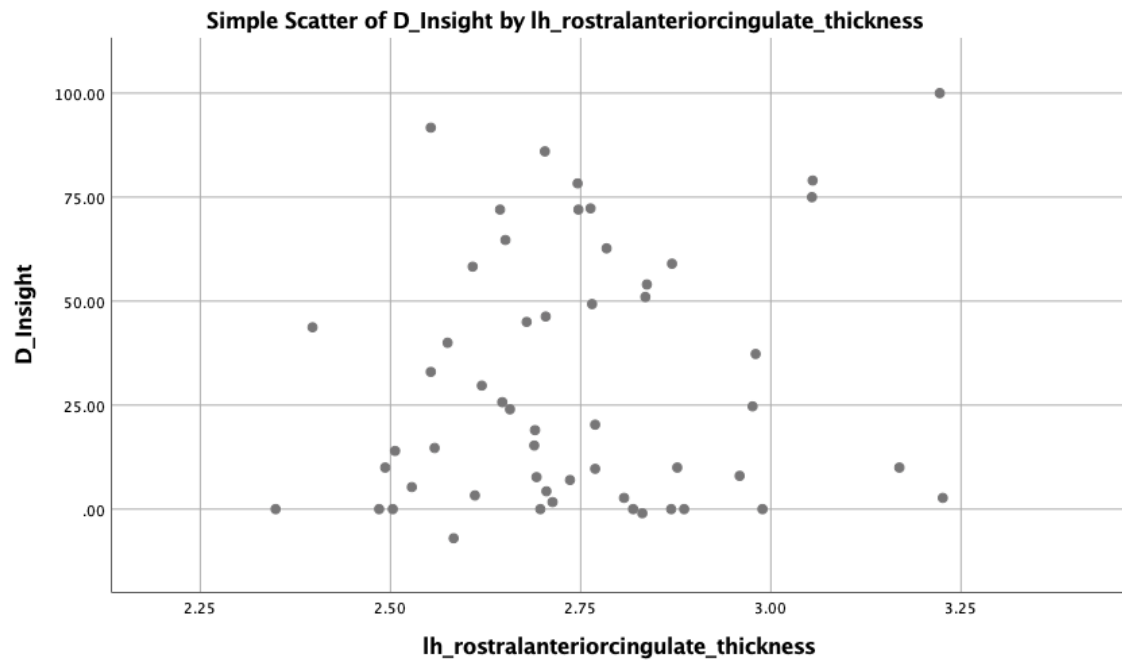

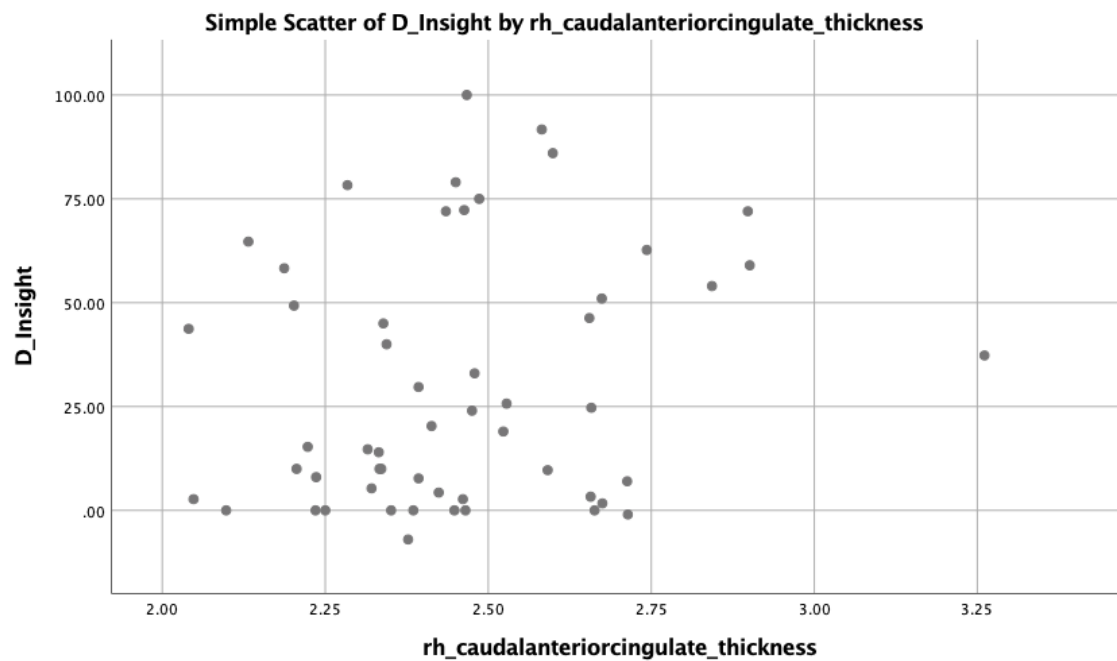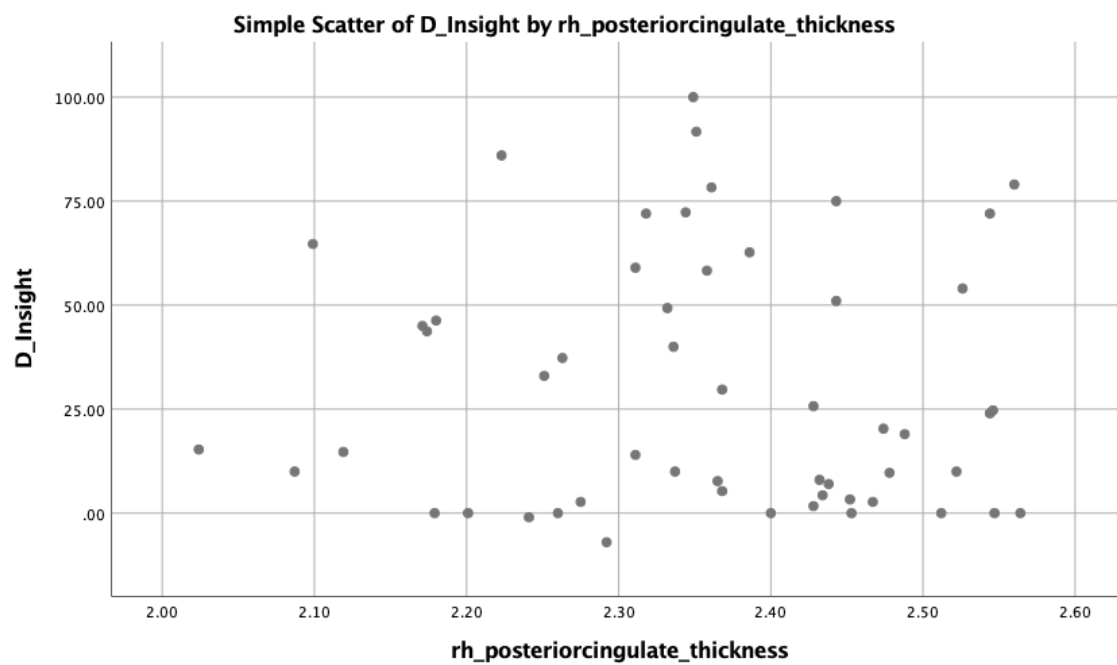

Supplement: Supplementary file 1 [file biomedicines-08-00034-s001.pdf]
